# The Effects of *Lactobacillus plantarum*, *Bacillus subtilis*, a Lignocellulolytic Enzyme System, and Their Combination on the Fermentation Profiles, Chemical Composition, Bacterial Community, and In Situ Rumen Digestion of Fresh Waxy Corn Stalk Silage

**DOI:** 10.3390/ani14233442

**Published:** 2024-11-28

**Authors:** Jiaqi Su, Ye Xue, Kunlun Zhang, Zihan Liu, Jingyi Lv, Qi Yang, Zhongqiu Li, Chunlong Liu, Hangshu Xin

**Affiliations:** 1 College of Animal Sciences and Technology, Northeast Agricultural University, Harbin 150030, China; sjq18393841697@163.com (J.S.); xueye422@163.com (Y.X.); zkl1185@163.com (K.Z.); neau13244537135@163.com (Z.L.); 18846440487@163.com (J.L.); qi_qyang@139.com (Q.Y.); 2 Northeast Institute of Geography and Agroecology, Chinese Academy of Sciences, Harbin 150081, China; 3 Key Laboratory of Combining Farming and Animal Husbandry, Ministry of Agriculture and Rural Affairs, Institute of Animal Husbandry, Heilongjiang Academy of Agricultural Sciences, Harbin 150086, China; lizhongqiu1974@163.com

**Keywords:** *Lactobacillus plantarum*, *Bacillus subtilis*, cellulase, silage, fresh waxy corn stalk

## Abstract

The objective of this study was to assess the impact of *Lactobacillus plantarum*, *Bacillus subtilis*, and a lignocellulolytic enzyme system on the nutritional value, fermentation profiles, rumen digestion, and bacterial community of fresh waxy corn stalk silage. Fresh waxy corn stalks harvested after 90 days of growth were treated with no additives (CON), compound multiple lactobacilli (ML, comprising *Lactobacillus plantarum* at 1.0 × 10^5^ cfu/g fresh weight and *Bacillus subtilis* at 1.0 × 10^5^ cfu/g fresh weight), a lignocellulolytic enzyme system (LE, at 500 g/t), and a combination of the lignocellulolytic enzyme system and multiple lactobacilli (MLLE). Throughout the fermentation process, the contents of dry matter (DM) and water-soluble carbohydrates (WSC), as well as the counts of lactic acid bacteria, yeast, and mold, continuously decreased with the extension of the fermentation time. In contrast, the concentrations of acetic acid, propionic acid, and ammonia N progressively increased over time. Notably, the pH initially decreased and then increased as fermentation progressed, while the lactic acid concentration first increased and then decreased with the extension of the fermentation time. After 45 days of fermentation, the DM contents of LE and MLLE were significantly lower than that of CON, while the WSC content of MLLE was significantly higher than that of CON. LE most effectively increased the ED of DM and neutral detergent fiber (NDF). Compared to ML and LE, MLLE showed a greater increase in the “a” value of DM and NDF. The DM content in LE and MLLE was lower than in CON, with a higher WSC content. Both ML and MLLE produced more lactic acid than CON and LE, resulting in a lower pH in these groups. Additionally, the acetic acid content and ammonia N content were lower in ML and MLLE compared to CON and LE. The counts of lactic acid bacteria, yeast, and mold declined over the fermentation period, dropping below detectable levels on the seventh and fifth days, respectively. Relative to CON, the three additives reduced the relative abundance of *Weissella* and *Klebsiella* and increased that of *Lactobacillus*. In conclusion, LE improved the nutritional value and rumen digestion of the silage, ML enhanced its fermentation profiles and bacterial community, and MLLE significantly improved the nutritional value, rumen digestion characteristics, fermentation profiles, and bacterial community of the fresh waxy corn stalk silage.

## Simple Summary

The black soil in Northeast China is one of the three largest black soil regions in the world. While producing large quantities of fresh waxy corn every year, this region also generates a significant amount of fresh waxy corn stalk. These high-sugar stalks not only hinder planting for the following year but also pose environmental pollution risks. A feasible solution to these issues is converting the fresh waxy corn stalks into ruminant feed through biological fermentation. In this study, we used three different silage additives to ferment fresh waxy corn stalks and compared their effects on the chemical composition, fermentation characteristics, ruminal degradability, culturable microorganisms, and bacterial community structure of the stalk. This study fills the data gap on the feed value of fresh waxy corn stalks and provides theoretical support for its application in ruminant production.

## 1. Introduction

Originating from China, fresh waxy corn stands as a distinguished variety of fresh corn [1]. The unique waxy gene responsible for the grain’s composition leads to an endosperm that is predominantly made up of amylopectin [1,2]. This specific composition bestows a distinct sticky texture upon the corn, a trait that has been highly prized in Chinese culinary traditions for centuries. Owing to these desirable characteristics, fresh waxy corn has achieved widespread acclaim and is extensively cultivated across China. In the year 2022, the total area dedicated to fresh corn cultivation in China was recorded at approximately 1.46 million hectares, with fresh waxy corn accounting for about 800,000 hectares.

The stalk of fresh corn, which is a byproduct obtained after harvesting of the ears, accounts for approximately 75% of the entire plant’s dry matter [3]. The moisture content of fresh waxy corn stalks is between 80% and 85%, which exceeds the recommended moisture range for optimal silage materials (65–70%). Fresh waxy corn stalks harvested at the 25% milk-line stage (early milk-line stage) are characterized by a higher sugar content and reduced lignification compared to conventional corn stalks harvested at the 100% milk-line stage (dent stage). Although the elevated sugar levels in fresh waxy corn stalks are advantageous for silage production, the challenges lie in their high fiber content and reduced digestibility, which are less than ideal for livestock nutrition. Furthermore, the typical harvesting period for fresh waxy corn stalks in Northeast China aligns with the rainy season, leading to an increased moisture content. Such conditions are conducive to *Clostridium* fermentation during the ensiling process, which can result in significant nutrient loss [4]. Consequently, the selection of appropriate additives becomes essential to enhance the fermentation profile of the silage and to improve the digestibility of the stalks for livestock purposes.

Cellulase, a widely utilized silage additive, can decompose structural carbohydrates such as cellulose into water-soluble carbohydrates [5]. This transformation not only provides substrates for the fermentation process of lactic acid bacteria but also disrupts the fibrous architecture, significantly enhancing the digestibility of silage feeds within the rumen [6]. For a long time, the exploration and assessment of cellulose degradation mechanisms predominantly focused on highly purified cellulose substrates. Specifically, within the realm of animal feed, enzyme activity assessments have been narrowly confined to disaccharide model substrates, thus overlooking the intricate composition and structural peculiarities of natural lignocellulose and their impact on enzymatic hydrolysis. In nature, the degrading enzymes secreted by both fungi and bacteria are complex enzyme systems rather than single enzyme components [7]. These different types of enzymes and enzyme components have a high degree of synergistic action during the degradation process [8]. Therefore, to improve the efficiency of fiber degradation in feed, it is essential to fully consider the specificity of the fiber and the integrity of the enzyme system. The substrate-specific lignocellulosic saccharification enzyme system used in this experiment is a set of degradation enzyme systems specifically developed based on the characteristics of the fiber in the feed, which is fully applicable to the current fiber characteristics of the feed and has the characteristics of being trace and highly efficient.

*Lactobacillus plantarum*, a homofermentative lactic acid bacterial strain, swiftly generates lactic acid, lowering the pH and suppressing spoilage microorganisms, which, in turn, enhances the fermentation quality of silage [3]. *Bacillus subtilis*, another silage additive, has been demonstrated to elevate the concentration of lactic acid and to improve the in vitro digestibility of organic matter, either alone or in synergy with *Lactobacillus plantarum* in corn silage [9]. Additionally, this combination has proven effective in diminishing yeasts and molds within the silage [9]. To date, research on the application of *Lactobacillus plantarum*, *Bacillus subtilis*, and cellulase aimed at enhancing silage quality and biodegradation behaviors has primarily concentrated on whole-plant corn, alfalfa, sweet sorghum, and certain herbage byproducts like Pennisetum sinese [10,11,12,13].

Therefore, this study aimed to investigate the effects of a lignocellulolytic enzyme system alongside combinations of *Bacillus subtilis* and *Lactobacillus plantarum* on the fermentation profiles, chemical composition, bacterial community, and rumen in situ degradability of fresh waxy corn stalk silage. The ultimate goal was to identify an efficient strategy for the judicious utilization of fresh waxy corn stalks.

## 2. Materials and Methods

### 2.1. Preparation of Corn Stalk Silage

The fresh waxy corn stalks used in this study were cultivated in Suihua City, located in Heilongjiang Province, China (46°58′ N, 126°14′ E). After the fresh waxy corn grains reached the 25% milk-line stage, they were manually harvested. On the following day, the fresh waxy corn stalks were harvested using a forage harvester, leaving a stubble height of 15–20 cm. Subsequently, the stalks were processed into 2–3 cm particles using a forage cutter (Sh-2000, Shanghai Donxe Industrial Co., Ltd., Shanghai, China). Following this, the uniformly chopped corn stalks were thoroughly mixed and then randomly distributed into 168 separate piles. Each pile was stored in a polyethylene film bag.

The experimental treatments were delineated as follows: (i) The control group (CON; without any additive). (ii) A blend of multiple lactobacilli strains (ML; Northeast Institute of Geography and Agroecology, Chinese Academy of Sciences, Harbin, China), including *Lactobacillus plantarum* (5.0 × 10^5^ cfu/g FW) and *Bacillus subtilis* (5.0 × 10^5^ cfu/g FW) (fresh weight; FW). (iii) A lignocellulolytic enzyme formulation (LE; also from Northeast Institute of Geography and Agroecology, Chinese Academy of Sciences, Harbin, China) applied at 500 g/t FW; this formulation mainly contains enzymes such as endoglucanase, exoglucanase, β-glucosidase, xylanase, amylase, and protease; the respective activities of these enzymes—filter paper enzyme, CMC enzyme, xylanase, exonuclease, β-glucosidase, amylase, and protease—were measured as 715.6 IU/g, 33,913.6 IU/g, 8340.7 IU/g, 3510.6 IU/g, 10,759 IU/g, 560 IU/g, and 280 IU/g, respectively. (iv) A combination of ML and LE (MLLE). Each polyethylene film bag (40 × 45 cm; 0.19 mm thickness) was filled with 1500 g of stalks. Based on the treatment group, the corresponding additive was added. In the ML, LE, and MLLE groups, the additives were dissolved in 10 mL of sterile water and evenly sprayed onto 1500 g of stalks. For the CON group, 10 mL of sterile water was evenly sprayed onto the stalks. Subsequently, a vacuum sealer (Maige Automation Equipment Co., Ltd., Qingdao, China) was employed to extract the air from the bags. The bags were opened after ensiling periods of 1, 3, 5, 7, 15, 30, and 45 days. Six bags were prepared as replicates for each treatment at each timepoint, totaling 168 bags in this study. Throughout the ensiling process, the bags were maintained at room temperature (15–25 °C).

### 2.2. Chemical Analysis

After a 45-day ensiling period, fresh waxy corn stalk silage samples were taken out from each polyethylene film bag, dried at 65 °C for 72 h, and then ground in a Wiley mill with a 1 mm screen for further chemical analysis. The dry matter (DM, method 925.40), crude protein (CP, method 2001.11), ether extract (EE, method 920.39), and ash (ash, method 942.05) of the fresh waxy corn stalks were measured according to AOAC [14]. The content of starch was determined using a starch content kit (Nanjing Jiancheng Biological Engineering Research Institute, Nanjing, China). The contents of neutral detergent fiber (NDF), acid detergent fiber (ADF), and acid detergent lignin (ADL) were analyzed using the method described by Van Soest [15]. Heat-stable alpha-amylase was used during the NDF analysis process to eliminate the starch effects. The content of water-soluble carbohydrates (WSC) was determined by colorimetry after reacting with anthrone reagent, following the method proposed by Li [13].

### 2.3. In Situ Rumen Digehstion

Stalks fermented for 45 days were used for in situ rumen digestion. The in situ rumen digestion of DM and NDF for all of the silage samples was determined in three Holstein cows (BW = 613 ± 10.2 kg, mean ± SD) with a rumen fistula, following the method described by Lv [16]. Each Holstein cow’s daily diet included 1 kg of sugar beet granules, 4 kg of dried oat grass, 2.4 kg of dried alfalfa grass (CP = 182.31 g/kg DM; NDF = 403.56 g/kg DM; ADF = 360.17 g/kg DM), and 2.3 kg of concentrate mixture (all three cows were six years old and are in the dry period, with their nutritional levels maintained at a sustaining state). The composition of the concentrate mixture per kilogram was 0.465 kg of corn, 0.11 kg of regular soybean meal, 0.12 kg of corn germ meal, 0.13 kg of non-sprayed germ meal, 0.035 kg of limestone powder, and 0.09 kg of corn distillers’ grains. Feeding was carried out daily at 5:00 and 17:00, and the cows had ad libitum access to water throughout the experimental period. Fresh waxy corn stalk silage used as samples was dried at 65 °C and ground to pass through a 2 mm screen using a Wiley mill (Arthur H. Thomas Co., Swedesboro, NJ, USA). Then, 2 g of the sample was weighed, put into nylon bags (80 × 150 mm, 38–40 µm pore size; Beijing Yiniuniu Information Technology Research Center (Limited Partnership), Beijing, China), and then tightly sealed with rubber bands. Prior to in situ rumen digestion, all nylon bags containing the samples were inserted into a larger mesh bag (36 × 42 cm) with a weight (2 kg) to ensure the placement below the particular mat layer in the ventral sac of the rumen. Ten timepoints were established: 0, 2, 4, 8, 12, 16, 24, 36, 48, and 72 hours. For each treatment, three replicates were set at each timepoint, with these replicates placed in the rumens of three different cows. The nylon bags were inserted in batches and removed together. After rumen incubation, all nylon bags were taken out, washed immediately with cold water, and then placed in an oven at 65 °C for 48 h before being weighed. The residues were sieved with a 1 mm mesh and transferred into a Ziplock bags. Finally, the feed residue samples were analyzed for DM and NDF [17]. The in situ rumen digestion experiment was conducted twice to ensure the accuracy and consistency of the results.

The fresh waxy stalks’ in situ degradation constants for DM and NDF were estimated using the formula described by Ørskov and McDonald [18]:

P = a + b (1 − e^−ct^)

where P is the rate of disappearance at time t (h), a is the rapidly degradable fraction, b is the potentially degradable fraction, and c is the rate of b.

The effective degradability (ED) of the silage samples was calculated as follows:

ED = a + bc/(c + kp)

where a, b, and c are constants, as described above. The pass rate (kp) of the corn stalk silage is assumed to be 45 g/kg h^−1^ [19].

### 2.4. Analyses of Fermentation Profiles and Microbial Counts

A 10 g sample of fresh waxy corn stalk silage was mixed with 90 mL of distilled water and refrigerated at 4 °C for 24 h. The silage samples were filtered using four layers of medical gauze, and the resulting filtrate was used for pH measurement with a pH meter (Sartorius basic pH meter, Göttingen, Germany). A 5 mL portion of the filtrate was transferred to a 10 mL centrifuge tube and centrifuged at 4 °C and 10,000× *g* for 15 min using a low-temperature high-speed centrifuge. The supernatant was then collected for the determination of ammonia nitrogen, lactic acid, and volatile fatty acid contents [20]. Lactic acids in the filtrate were quantified using high-performance liquid chromatography (Carbomix H-NP5 column, 55 °C, 2.5 mM H_2_SO_4_, 0.5 mL/min), following the method described by Lv [16]. The concentrations of acetic acid and propionic acid were determined using a gas chromatograph (GC-2010 Gas Chromatograph, Shimadzu Corporation, Kyoto, Japan) equipped with a gas chromatography column (Agilent J&W HP-INNOWax), with the length and diameter being 30 cm and 0.25 mm, respectively.

A 10 g sample of fresh waxy corn stalk silage was homogenized in 90 mL of sterilized saline water for 2 h, and the homogenate was diluted into different gradients and spread onto agar plates to facilitate bacterial growth. The homogenate, coated on Man Rogosa–Sharpe agar (MRS) medium (Sinopharm Chemical Reagent Co., Ltd., Shanghai, China), was placed inside a microbial anaerobic culture box (Mitsubishi Gas Chemical Company, Inc., Tokyo Metropolis, Japan) and transferred to a 37 °C incubator (SPX-250 BIII, Boxun., Shanghai, China) for 48 h to enumerate the lactic acid bacteria (LAB). For the purpose of counting mold and yeast, the homogenate coated on Rose Bengal Chloramphenicol Agar (RBC) medium (Sinopharm Chemical Reagent Co., Ltd., Shanghai, China) was incubated in a 30 °C incubator (Fx303-1, Shanghai Shuli Instrumentation Co., Ltd., Shanghai, China) for 48 h. The colony count was subsequently converted to represent the number of viable bacteria per gram of fresh weight (cfu/g FW) for statistical analysis.

### 2.5. Microbial Diversity Analysis

The V3-V4 region of bacterial 16S rRNA genes was profiled using the 314 F (5′-CCTAYGGGRBGCASCAG-3′) and 806 R (5′-GGACTACNNGGGTATATAAT-3′) primers. The amplicons were then sequenced (2 × 250 bp) using the Nova PE250 on the NovaSeq 6000 platform (Novogene, Tianjin, China). The sequence data were analyzed using Quantitative Insights into Microbial Ecology 2 (QIIME2, version 2023.02). Amplicon sequence variants were generated using the DADA2 workflow to eliminate the barcodes, primers, and low-quality sequences. Taxonomic classification was performed using the SILVA database (SILVA Release 138), based on 99% sequence similarity.

### 2.6. Statistical Analyses

Partial data in this experiment were analyzed using SAS software (version 9.4, SAS Institute Inc., Cary, NC, USA). The NLIN procedure was employed to estimate the in situ degradation kinetics parameters, including a, b, c, and ED. Based on the model Y_ij_ = µ + T_i_ + e_j_, an ANOVA procedure was performed to analyze the differences in the chemical composition and in situ rumen degradation kinetics parameters of four varieties of fresh waxy corn stalk silage, where Y_ij_ is the dependent variable, µ is the overall mean, T_i_ is the treatment effect, and e_j_ is the error term. Significant differences were considered at *p* < 0.05. Using the model Y_ijk_ = µ + α_i_ + β_j_ + (α × β)_ij_ + e_ijk_, a two-way ANOVA was conducted to assess the effects of silage additives, silage days, and their interactions on fermentation parameters, microbial community numbers, DM content, and WSC content. In this model, Y_ijk_ is the dependent variable, *μ* is the overall mean, α_i_ is the fixed effect of treatment, β_j_ is the effect of ensiling days, (α × β)_ij_ represents the interaction between treatment and ensiling days, and e_ijk_ is the residual error. Duncan’s method was used for multiple comparisons, with *p* < 0.05 regarded as significant differences.

A beta diversity analysis of microbial communities was conducted using principal component analysis (PCA) to identify and quantify the similarities and differences among the various samples. The silage’s relative abundance of bacterial taxa and alpha diversity were analyzed using the Kruskal–Wallis test and the Wilcoxon test in R (version 4.0.2; R Foundation for Statistical Computing, Vienna, Austria), and statistical significance was declared at *p* ≤ 0.05.

## 3. Results

### 3.1. Nutrient Compositions of Fresh Waxy Corn Stalk Silage After 45 Days of Ensiling

As shown in Table 1, fresh waxy corn stalks subjected to different treatments exhibited distinct nutrient compositions. The ash content of MLLE and LE was significantly higher than that of CON and ML (*p* < 0.05), with that of MLLE ranking as the highest among the four treatments. There was no significant difference in the CP (*p* = 0.10) and EE (*p* = 0.56) contents among the four treatments. In terms of NDF and ADF contents, CON exhibited significantly higher values compared to ML, LE, and MLLE (*p* < 0.05), with the content of MLLE being the lowest among the four treatments. While the ADL content of LE and MLLE was higher than that of CON and ML, no significant difference was observed between the different treatments (*p* = 0.16).

### 3.2. In Situ Ruminal Degradation of Fresh Waxy Corn Stalk Silage After 45 Days of Ensiling

Table 2 illustrates the in situ ruminal degradation kinetics of all silages. In terms of the in situ DM rumen digestion kinetics, all additives significantly increased the parameter “a”, when compared to CON, with MLLE exhibiting the most substantial increase, reaching 95.33% (*p* < 0.05). Regarding the parameter “b”, there was no significant difference between ML and LE compared to CON, but MLLE demonstrated a significant decrease in comparison (*p* < 0.05). The “c” value was increased by all three additives in comparison to CON, with LE showing the highest increase at 9.53%. However, there was no significant difference observed among the four treatments. Furthermore, the three additives significantly increased the ED value when compared to CON (*p* < 0.05), with LE showing the highest increase at 7.23%, followed by MLLE at 5.41%.

The findings suggest that all three additives significantly raised the value of parameter “a” in the NDF in situ rumen digestion kinetics (*p* < 0.05), with MLLE showing the most substantial increase. When compared to CON, ML and MLLE did not bring about significant changes in the values of parameters “b” and ED, while LE notably increased both “b” and ED (*p* < 0.05). In addition, in comparison to CON, none of the three additives resulted in significant alterations in the value of parameter “c”.

### 3.3. Fermentation Profile, Water-Soluble Carbohydrate and Microbial Populations of Fresh Waxy Stalk Silage During Ensiling Time (1, 3, 5, 7, 15, 30, and 45 Days)

**Figure 1:**
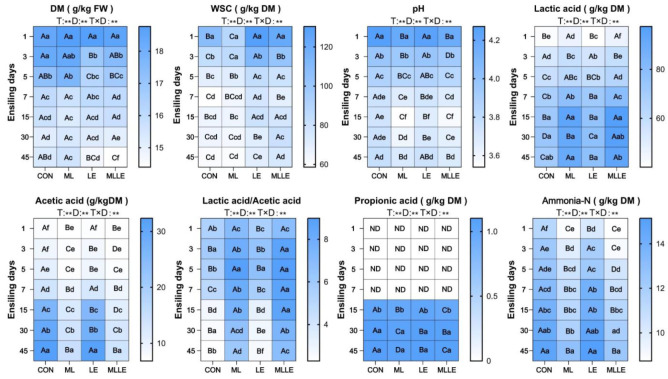
Effects of different treatments on DM, WSC, and fermentation profiles of fresh waxy corn stalk silage during ensiling. WSC, water-soluble carbohydrate; FW, fresh weight, CON, control; ML, *Lactobacillus plantarum* at 1.0 × 10^5^ cfu/g fresh weight and *Bacillus subtilis* at 1.0 × 10^5^ cfu/g fresh weight; LE, lignocellulolytic enzyme system at 500 g/t fresh weight; MLLE, *Lactobacillus plantarum* at 1.0 × 10^5^ cfu/g fresh weight, *Bacillus subtilis* at 1.0 × 10^5^ cfu/g fresh weight and lignocellulolytic enzyme system at 500 g/t fresh weight. T, treatment; D, days of fermentation; T×D, interaction between treatment and fermentation time; ND, not detected. Different uppercase letters indicate significant differences among treatments on the same day; different lowercase letters indicate significant differences among days within the same treatment. “**” indicates *p* < 0.01.

Figure 1 illustrates the changes in fermentation profiles and WSC content in fresh waxy corn stalk silage over a 45-day fermentation period. The DM content gradually decreased over time. Notably, the DM content of fresh waxy corn stalks treated with LE was lower than that without LE (*p* < 0.05). Simultaneously, the WSC content decreased as the fermentation period was extended (*p* < 0.05). However, in contrast to the trend observed in DM content, the WSC content of fresh waxy corn stalks with LE was higher than that without LE (*p* < 0.05).

During the fermentation, the pH of all four treatments dropped below 4.0 on the third day and reached its lowest point on the fifteenth day, before gradually increasing to a stable level. On the 45th day, the pH of fresh waxy corn stalks with LA was lower than that without LA (*p* < 0.05). Additionally, on the 45th day, the lactic acid content of ML, LE, and MLLE was significantly higher than that of CON (*p* < 0.05), with ML showing a more pronounced increase than LE. As time progressed, the acetic acid content increased gradually in all treatments. On the 45th day, the acetic acid content of ML and MLLE was significantly lower than that of CON and LE (*p* < 0.05). The lactic acid/acetic acid ratio in the silage samples initially increased and then decreased as the time was extended. On the 45th day of fermentation, the lactic acid/acetic acid ratio exceeded 6.0 in ML and MLLE, while it remained below 3.0 in CON and CE.

Propionic acid was not detected in the initial seven days of fermentation. Although a notable difference in propionic acid content among the different treatments was observed at the end of fermentation (*p* < 0.05), the extent of this difference was not substantial. Butyric acid remained undetected throughout the entire fermentation process. The content of ammonia N increased steadily during the fermentation. On the 45th day of fermentation, the ammonia N content in CON and LE was significantly higher than that in ML and MLLE (*p* < 0.05).

**Figure 2:**
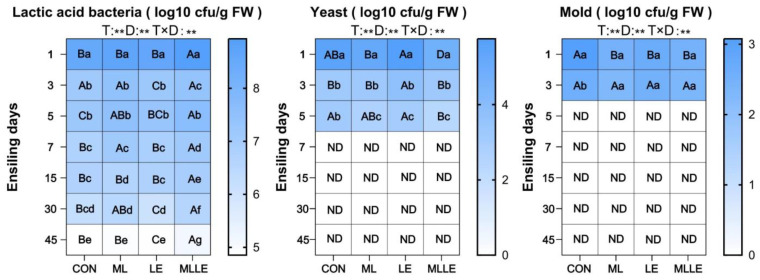
Effects of different treatments on the counts of lactic acid bacteria, yeast, and mold during the fermentation of fresh waxy corn stalk silage. FW, fresh weight; CON, control; ML, *Lactobacillus plantarum* at 1.0 × 10^5^ cfu/g fresh weight and *Bacillus subtilis* at 1.0 × 10^5^ cfu/g fresh weight; LE, lignocellulolytic enzyme system at 500 g/t fresh weight; MLLE, *Lactobacillus plantarum* at 1.0 × 10^5^ cfu/g fresh weight, *Bacillus subtilis* at 1.0 × 10^5^ cfu/g fresh weight and lignocellulolytic enzyme system at 500 g/t fresh weight. T, treatment; D, days of fermentation; T×D, interaction between treatment and fermentation time; ND, not detected. Different uppercase letters indicate significant differences among treatments on the same day; different lowercase letters indicate significant differences among days within the same treatment. “**” indicates *p* < 0.01.

Figure 2 shows that counts of lactic acid bacteria, yeast, and mold exhibited a consistent decrease as the ensiling days progressed. In all treatments, yeast was undetectable on the 7th day, while mold was not detected on the 5th day during fermentation.

### 3.4. The Effects of Three Additives on the Bacterial Community of Fresh Waxy Corn Stalk Silage After 45 Days of Ensiling

Table 3 shows the effects of three additives on the bacterial alpha diversity index. Within the studied microbial communities, the Shannon index in the LE treatment group was lower compared to that in the CON group, although this variance was not statistically significant. In contrast, the Shannon index in the ML and MLLE treatment groups was significantly lower than that in the CON group (*p* < 0.05), with the most pronounced disparity observed between the MLLE and CON groups. Additionally, the richness, Chao1, and Ace indices were significantly higher in the CON group than in the ML, LE, and MLLE groups (*p* < 0.05). The most substantial gaps in the richness, Chao1, and Ace indices were observed between the MLLE and CON groups.

**Figure 3:**
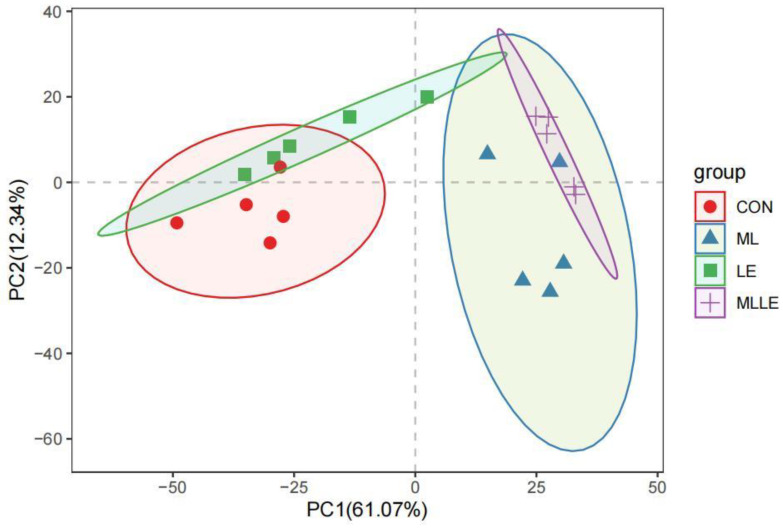
Principal component analysis of the bacterial community in fresh waxy corn stalk silage ensiled with four additives after 45 days. CON, control; ML, *Lactobacillus plantarum* at 1.0 × 10^5^ cfu/g fresh weight and *Bacillus subtilis* at 1.0 × 10^5^ cfu/g fresh weight; LE, lignocellulolytic enzyme system at 500 g/t fresh weight; MLLE, *Lactobacillus plantarum* at 1.0 × 10^5^ cfu/g fresh weight, *Bacillus subtilis* at 1.0 × 10^5^ cfu/g fresh weight, and lignocellulolytic enzyme system at 500 g/t fresh weight.

In Figure 3, the principal component analysis revealed that the CON, ML, LE, and MLLE treatments were distinctly positioned in the third, fourth, first, and second quadrants, respectively. This distribution indicates that different additives exerted a significant impact on the microbial communities of fresh waxy corn stalk silage after 45 days of anaerobic fermentation.

**Figure 4:**
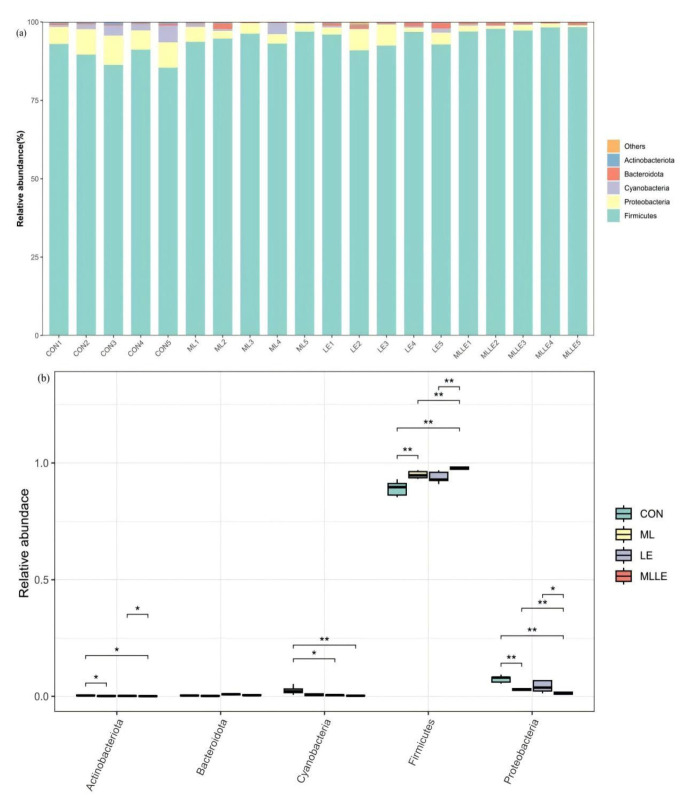
The effects of three additives on the bacterial community composition at the phylum level in fresh waxy corn stalk silage after 45 days of ensiling ((**a**) shows the relative abundance of different bacterial genera in each sample, while (**b**) presents the Kruskal-Wallis and Wilcoxon analysis results of the main genera between different treatments). CON, control; ML, *Lactobacillus plantarum* at 1.0 × 10^5^ cfu/g fresh weight and *Bacillus subtilis* at 1.0 × 10^5^ cfu/g fresh weight; LE, lignocellulolytic enzyme system at 500 g/t fresh weight; MLLE, *Lactobacillus plantarum* at 1.0 × 10^5^ cfu/g fresh weight, *Bacillus subtilis* at 1.0 × 10^5^ cfu/g fresh weight, and lignocellulolytic enzyme system at 500 g/t fresh weight. *, *p* < 0.05; **, *p* < 0.01.

In Figure 4, the bacterial community composition at the phylum level in fresh waxy corn stalk silage subjected to various treatments is depicted, along with their relative abundance differences. The five most abundant phyla, ranked in descending order, are Firmicutes, Proteobacteria, Cyanobacteria, Bacteroidota, and Actinobacteriota. Among the four treatments examined, the relative abundance of Firmicutes in the MLLE treatment was higher than in the other three treatments (*p* < 0.05). The descending order of Proteobacteria’s relative abundance across the four treatments was CON, LE, ML, and MLLE. Notably, the relative abundance of Proteobacteria in CON was significantly greater than that in the LE and MLLE treatments (*p* < 0.05), while LE exhibited a significantly higher abundance compared to MLLE (*p* < 0.05). Additionally, the relative abundance of Cyanobacteria in CON was significantly higher than that in both LE and MLLE (*p* < 0.05).

**Figure 5:**
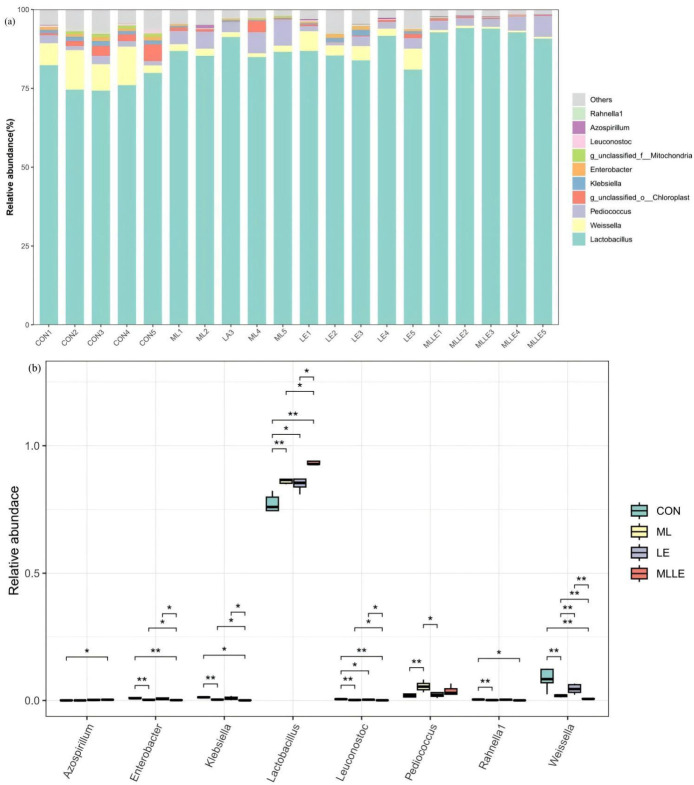
The effects of three additives on the bacterial community composition at the genus level in fresh waxy corn stalk silage after 45 days of ensiling ((**a**) shows the relative abundance of different phyla in each sample, while (**b**) presents the Kruskal-Wallis and Wilcoxon analysis results of the main phyla between different treatments). CON, control; ML, *Lactobacillus plantarum* at 1.0 × 10^5^ cfu/g fresh weight and *Bacillus subtilis* at 1.0 × 10^5^ cfu/g fresh weight; LE, lignocellulolytic enzyme system at 500 g/t fresh weight; MLLE, *Lactobacillus plantarum* at 1.0 × 10^5^ cfu/g fresh weight, *Bacillus subtilis* at 1.0 × 10^5^ cfu/g fresh weight, and lignocellulolytic enzyme system at 500 g/t fresh weight. *, *p* < 0.05; **, *p* < 0.01.

Figure 5 illustrates the top ten genera in terms of relative abundance at the genus level and highlights the differences in relative abundance among these known genera. *Lactobacillus* emerged as the dominant genus across all four treatments. In comparison to the CON treatment, the relative abundance of *Lactobacillus* was significantly higher in the other three treatments (*p* < 0.05). Regarding the relative abundance of *Weissella*, both the ML and MLLE treatments exhibited a significantly lower abundance than CON and LE (*p* < 0.05). The relative abundance of *Pediococcus* in the ML treatment was notably higher than that in both CON and LE (*p* < 0.05). The introduction of multiple lactobacilli strains markedly reduced the relative abundance of *Klebsiella* in fresh waxy corn stalks; specifically, the relative abundance of *Klebsiella* in the ML and MLLE treatments was significantly lower than in the CON treatment (*p* < 0.05). Regarding the relative abundance of *Enterobacter*, the CON treatment displayed a significantly higher abundance than ML and MLLE (*p* < 0.05), while the abundance in MLLE was significantly lower than in CON, LE, and ML (*p* < 0.05).

## 4. Discussion

### 4.1. Nutrient Compositions of Fresh Waxy Corn Stalk Silage After 45 Days of Ensiling

High-moisture and nutrient-rich silage materials provide an ideal liquid environment and abundant fermentable substrates for the growth of lactic acid bacteria and other microorganisms, promoting their rapid proliferation. Simultaneously, large amounts of soluble sugars, soluble fibers, peptides, and other readily soluble nutrients are converted into lactic acid, volatile fatty acids, and ammonia nitrogen. The lignocellulolytic enzyme system added in the LE and MLLE treatments further aided in converting cellulose into WSC, ultimately leading to an excessive conversion of nutrients into lactic acid, volatile fatty acids, and ammonia nitrogen, resulting in a reduction in DM content [4]. The impact of the different treatments on CP content was not significant, primarily due to the rapid rate of fermentation. Maintaining a pH below 4.2 can effectively inhibit the growth of harmful microorganisms, thereby reducing the loss of protein and nutrients in silage [21]. By the third day, the pH levels in all treatments were below 4.0, preventing excessive decomposition of protein into ammonia nitrogen. Supporting this, research by de Almeida Rufino indicated that lactic acid bacteria additives do not significantly affect the CP content of well-fermented silage [22].

Our results demonstrated that the inclusion of the three additives effectively reduced the contents of NDF and ADF. This aligns with the findings of Xu, who reported that adding *Lactobacillus plantarum* alone or in combination with cellulase, significantly reduced the NDF and ADF contents after 90 days [23]. In our study, MLLE resulted in a greater reduction of in NDF and ADF than the treatments with ML or LE alone. This increase was likely the result of the synergistic interaction between *Bacillus subtilis*, *Lactobacillus plantarum*, and the lignocellulolytic enzyme system. The lignocellulolytic enzyme system played a crucial role in deconstructing cellulose into water-soluble carbohydrates, thereby furnishing the necessary substrates for the growth and propagation of *Bacillus subtilis* and *Lactobacillus plantarum*. Concurrently, the cellulase enzymes produced by *Bacillus subtilis* and *Lactobacillus plantarum* contributed to further degradation of cellulose, augmenting the conversion process of cellulose into accessible nutrients. Similarly, Zhao and Li found that the combination of lactic acid bacteria and cellulase degrades structural carbohydrates more effectively than when added separately [13,24].

### 4.2. In Situ Ruminal Degradation of Fresh Waxy Corn Stalk Silage After 45 Days of Ensiling

The rumen digestion rate of DM and NDF in roughage is a crucial factor in determining the DMI of livestock, thereby serving as a significant indicator for assessing nutritional values [25]. In our study, the three additives effectively improved the “a” fraction and ED of DM and NDF. This enhancement could be attributed to the cellulase produced by *Bacillus subtilis* in ML and MLLE, which converts a portion of the cellulose and hemicellulose into water-soluble carbohydrates [5]. Furthermore, the lignocellulolytic enzyme system and cellulase from *Bacillus subtilis* broke down the cell wall structure of the stalk, increasing the surface area for bacterial attachment and thereby enhancing the rumen digestion rate of the silage. Initially, we hypothesized that the MLLE treatment would most significantly improve the rumen digestion rate of the silage. However, our results indicated that while the improvement with MLLE was greater than with ML, it was lower than with LE. We speculate that this may be due to the rapid reduction in pH during the early stages of silage treatment with MLLE, potentially inhibiting cellulase activity, and thus, reducing its efficacy in enhancing the rumen digestion rate of silage.

### 4.3. Fermentation Profiles, Water-Soluble Carbohydrate, and Microbial Populations of Fresh Waxy Stalk Silage During Ensiling Time (1, 3, 5, 7, 15, 30, and 45 Days)

Throughout the ensilage fermentation cycle, no significant differences were observed in DM content among the four silage treatments on days 1, 7, 15, and 30 of fermentation. However, on days 3, 5, and 45, although statistical differences were present among the four silage treatments, the numerical differences were minimal. This could be attributed to the lower DM content in the fermentation materials, rapid fermentation rate, and well-preserved silage [4]. Due to the high moisture content of silage materials (above 75% FM) and the elevated levels of WSC, which provide an ideal liquid environment and ample substrates for the proliferation of lactic acid bacteria and lactic acid production, the lactic acid content in fresh waxy corn stalks exceeded 40 g/kg DM after one day of fermentation, reducing the pH to below 4.30 [21]. As is well known, WSC constitutes the primary substrate during silage fermentation. When the WSC content is low, the population of lactic acid bacteria decreases, potentially leading to the proliferation of harmful bacteria such as *Clostridium* [26]. Thus, maintaining a sufficient WSC content is crucial for silage preservation. Cellulase can convert structural carbohydrates into WSC, resulting in significantly higher WSC content in the LE and MLLE treatments compared to the other two treatments. High-quality silage raw materials should have a WSC content higher than 60 g/kg and a lactic acid bacteria content exceeding 1 × 10^5^ cfu/g fresh weight [27]. Therefore, quality silage can also be produced from fresh waxy corn stalks through natural fermentation.

The pH values of all four treatments were below 4.0 on the third day, which may be attributable to the DM content being significantly lower than the recommended range of 32–35% [28]. Queiroz reported that silage with a low DM content exhibited efficient fermentation and a rapid decline in pH [29]. Guo noted that a pH lower than 4.5 effectively inhibited the growth of putrefactive bacteria, and a pH below 4.2 is considered to be an important indicator of well-preserved silage [30]. The addition of *Lactobacillus plantarum* and *Bacillus subtilis* in MLLE and ML resulted in a faster decrease in pH. By the second day of ensiling, the pH levels of the ML and MLLE treatments were below 4.2. This is primarily because *Lactobacillus plantarum*, as a homofermentative lactic acid bacterium, can efficiently convert WSC into lactic acid compared to heterofermentative lactic acid bacteria, leading to a rapid decrease in pH in the early stages of silage [10]. Ding also indicated that adding homofermentative lactic acid bacteria to rice stalks could effectively accelerate the rate of pH decrease in the early stage of silage fermentation. In this study, the lactate content in the LE treatment was higher than that in the CON treatment but significantly lower than that in the ML and MLLE treatments, which might have been due to the addition of LE converting some fibers into WSC, thereby providing more substrates for lactic acid bacteria and resulting in more lactate production than in the CON treatment [31].

During the first seven days of ensiling, lactic acid bacteria exhibited their most vigorous activity, consuming a substantial amount of WSC, ranging from 28.2% to 46.0%, and rapidly producing lactic acid [32]. Under natural conditions, homofermentative lactic acid bacteria initially dominate the early stage of silage fermentation, characterized by an LA/AA ratio greater than 3.0.

Research by Ding indicated that adding cellulase enzymes to silage could reduce its pH and increased its lactic acid content but did not alter the fermentation type [31]. This is consistent with our findings for the CON and LE treatments. In the late fermentation stage under natural conditions, as the pH decreases, homotypic lactic acid bacteria are inhibited, paving the way for acid-tolerant heterotypic lactic acid bacteria to dominate the fermentation. These heterotypic bacteria also convert a portion of the lactic acid into acetic acid while producing lactic acid, leading to an LA/AA ratio of less than 3.0 in the later stages of silage, along with loss of nutrition [33]. The addition of *Lactobacillus plantarum* not only inhibited harmful microorganisms but also suppressed the growth of heterofermentative lactic acid bacteria, thereby changing the fermentation type and decreasing the loss of nutrition [3]. Laleye reported that *Lactobacillus plantarum* inhibited the growth and reproduction of various bacteria, including heterofermentative acid bacteria, through the release of antibacterial substances such as hydrogen peroxide and lactic acid during the fermentation process [34]. Our findings are consistent with those of Ding and Ma, indicating that CON and LE initially underwent homotypic fermentation, followed by heterotypic fermentation in the later stages [3,35]. In contrast, the ML and MLLE treatments maintained homotypic fermentation throughout the entire silage process. The low levels of ammonia N and propionic acid might be attributable to the rapid decline in pH during the early fermentation period, leading to the swift inhibition of harmful microorganisms such as Clostridium.

The population of lactic acid bacteria decreased over the course of fermentation—a finding that is consistent with the results of a recent study [4]. The rapid disappearance of yeasts and molds during early fermentation could be primarily attributed to the characteristics of the fresh waxy corn stalks, which included low DM content and high sugar content [35]. Silage materials with lower moisture content ferment more rapidly, and the abundance of soluble sugars provides sufficient substrates for rapid lactic acid fermentation [4]. Consequently, fresh waxy corn stalk produces a significant amount of lactic acid in the early stages of fermentation, which inhibits the proliferation of yeasts and molds. Additionally, on the first day of fermentation, the pH values for CON, ML, LE, and MLLE were 4.27, 4.19, 4.25, and 4.18, respectively. When the pH falls below 4.20, microorganisms, including lactic acid bacteria, are inhibited. Therefore, the population of lactic acid bacteria reached its highest level on the first day of fermentation.

### 4.4. The Effects of Three Additives on the Bacterial Community of Fresh Waxy Corn Stalk Silage After 45 Days of Ensiling

The changes observed in the Shannon, richness, Chao1, and Ace indices among the treatments in our study could be attributed to the fact that all three additives increased the lactic acid content, subsequently lowering the pH of the ensiled stalks. This acidic environment impeded microbial growth, with the ML and LE treatments exerting a synergistic effect that resulted in an even lower pH, thereby leading to a more pronounced inhibition of microbial activity [36].

After 45-day fermentation, the relative abundances of Firmicutes and Proteobacteria made them the dominant phyla in the silage, with Firmicutes being the most abundant, which was consistent with the observations in the studies of Mu and Yuan. Under anaerobic conditions, the Firmicutes phylum predominantly consists of acid-hydrolyzing microorganisms, including genera such as *Lactobacillus*, *Pediococcus*, *Weissella*, and *Leuconostoc* [5,37]. In contrast, the Proteobacteria phylum primarily encompasses organisms that decompose organic matter, featuring genera like *Klebsiella*, *Enterobacter*, *Leuconostoc*, *Azospirillum*, and *Rahnella*. Various microorganisms within the Firmicutes phylum can produce lactic acid, a process that subsequently lowers the environmental pH [4]. This reduction in pH serves as a mechanism to inhibit the growth and reproduction of microorganisms belonging to other phyla [5].

As reported by Ni, *Lactobacillus*, *Pediococcus*, *Weissella*, and *Leuconostoc* are recognized for their crucial roles in lactic acid fermentation during silage production [38]. Initially, *Weissella*, *Pediococcus*, and *Lactococcus* prevail during the early stages of silage fermentation. However, as the pH decreases in later stages, the dominant genera shift to *Lactobacilli*, known for their greater tolerance to low pH [39]. In our study, the relative abundance of *Lactobacillus* in the CON and LE treatments was significantly lower than in the MLLE and ML treatments. Conversely, the relative abundances of *Weissella* and *Pediococcus* were significantly higher in the CON group compared to the other three groups. The research conducted by Wang corroborates our findings, suggesting that the incorporation of cellulase could effectively enhance the relative abundance of Lactobacilli [26]. Our hypothesis posits that, within the MLLE, cellulase facilitates the conversion of fiber into soluble carbohydrates during fermentation. This enzymatic process enriches the substrate pool for lactic acid bacteria, consequently promoting their substantial proliferation during the initial stages of fermentation. However, the study by Zhang presented a contrasting viewpoint, as the addition of cellulase did not significantly impact the abundance of *Lactobacillus* in whole-plant corn silage [10]. In silage materials characterized by low soluble sugar content, cellulase plays a pivotal role in converting lignocellulose into soluble sugars. This conversion provides a substrate that is essential for the reproduction of lactic acid bacteria, thereby enhancing the relative abundance of *Lactobacillus*. However, in the case of whole-plant corn silage, which inherently contains high levels of soluble sugars, the availability of sugar does not significantly restrict the reproduction of lactic acid bacteria. Consequently, this might be one of the possible reasons for the unchanged relative abundance of Lactobacillus in silage samples treated with cellulase.

*Weissella*, a heterofermentative bacterium, is commonly found in untreated silage [4]. Research by Fusco demonstrated that various *Weissella* species could thrive in environments with a pH as low as 3.9 [40]. However, during the advanced stages of silage fermentation, the growth and reproduction of *Weissella* were significantly hindered by the reduced pH. Consequently, as silage fermentation progresses, the relative abundance of *Weissella* decreases. Studies indicated a notable reduction in the relative abundance of *Weissella* in the ML and MLLE treatments compared to CON and LE. This reduction may be associated with the accelerated pH decline observed in the ML and MLLE samples, particularly the lower pH recorded on the 45th day of fermentation.

*Klebsiella*, a genus within the Enterobacteriaceae family, is considered to be undesirable during the process of silage fermentation [4]. An important observation is the inverse relationship between silage fermentation profiles and the relative abundance of *Klebsiella* [10]. This study revealed a significantly higher relative abundance of *Klebsiella* in the CON and LE treatments compared to ML and MLLE. This suggests that the application of compound lactic acid bacteria exerts a substantial inhibitory effect on *Klebsiella* proliferation.

## 5. Conclusions

After 45 days of anaerobic fermentation, we found that, compared to CON and ML, LE and MLLE significantly improved the nutritional value of fresh waxy corn stalk silage. This was evident through reductions in NDF and ADF, along with enhanced rumen degradability. Throughout the fermentation process, both ML and MLLE increased the contents of lactic acid and WSC, as well as the relative abundance of Lactobacillus. At the same time, these treatments decreased the ammonia nitrogen content, pH, and the relative abundance of other bacterial genera, effectively altering the fermentation type of the silage. These results highlight that LE was effective in boosting the silage’s nutritional value, ML in enhancing its fermentation quality, and MLLE in improving both the nutritional value and fermentation quality of the silage. Therefore, incorporating MLLE into fresh waxy corn stalks presents an effective strategy for enhancing silage quality.

## Figures and Tables

**Table 1:** Nutrient composition of fresh waxy corn stalk silage after 45 days of ensiling.

Items	Treatment				SEM	*p*
	CON	ML	LE	MLLE		
Ash (g/kg DM)	89.09 ^C^	89.63 ^C^	94.16 ^B^	100.60 ^A^	0.595	<0.01
CP (g/kg DM)	40.20	41.06	40.32	41.33	0.354	0.10
EE (g/kg DM)	13.19	13.20	13.32	14.13	0.545	0.56
NDF (g/kg DM)	774.19 ^A^	751.19 ^B^	750.93 ^B^	703.28 ^C^	3.611	<0.01
ADF (g/kg DM)	480.15 ^A^	458.23 ^B^	461.61 ^B^	420.08 ^C^	2.495	<0.01
ADL (g/kg DM)	55.10	54.38	57.91	57.14	1.257	0.16

CON, control; ML, *Lactobacillus plantarum* at 1.0 × 10^5^ cfu/g fresh weight and *Bacillus subtilis* at 1.0 × 10^5^ cfu/g fresh weight; LE, lignocellulolytic enzyme system at 500 g/t fresh weight; MLLE, *Lactobacillus plantarum* at 1.0 × 10^5^ cfu/g fresh weight, *Bacillus subtilis* at 1.0 × 10^5^ cfu/g fresh weight, and lignocellulolytic enzyme system at 500 g/t fresh weight. Ash, crude ash; CP, crude protein; EE, ether extract; NDF, neutral detergent fiber; ADF, acid detergent fiber; ADL, acid detergent lignin. Means in a row with similar superscripts do not differ (*p* > 0.05); SEM, standard error of means.

**Table 2:** In situ ruminal degradation kinetics in fresh waxy corn stalk silage.

Items		Treatment				SEM	*p*
		CON	ML	LE	MLLE		
DM	a (g/kg)	78.67 ^C^	106.83 ^B^	112.00 ^B^	153.67 ^A^	3.253	<0.01
	b (g/kg)	544.50 ^A^	536.33 ^A^	556.33 ^A^	503.33 ^B^	8.506	<0.01
	c (g/kg h^−1^)	38.50 ^A^	40.17 ^A^	39.00 ^A^	42.17 ^A^	2.060	0.61
	ED (g/kg)	622.67 ^C^	642.50 ^BC^	667.67 ^A^	656.33 ^AB^	7.569	<0.01
NDF	a (g/kg)	76.67 ^B^	92.17 ^A^	94.33 ^A^	98.33 ^A^	3.409	<0.01
	b (g/kg)	563.67 ^B^	564.83 ^B^	593.83 ^A^	555.33 ^B^	8.091	0.02
	c (g/kg h^−1^)	40.00 ^A^	42.33 ^A^	38.17 ^A^	43.17 ^A^	2.010	0.31
	ED (g/kg)	640.00 ^B^	656.33 ^B^	687.67 ^A^	653.17 ^B^	7.348	<0.01

CON, control; ML, *Lactobacillus plantarum* at 1.0 × 10^5^ cfu/g fresh weight and *Bacillus subtilis* at 1.0 × 10^5^ cfu/g fresh weight; LE, lignocellulolytic enzyme system at 500 g/t fresh weight; MLLE, *Lactobacillus plantarum* at 1.0 × 10^5^ cfu/g fresh weight, *Bacillus subtilis* at 1.0 × 10^5^ cfu/g fresh weight, and lignocellulolytic enzyme system at 500 g/t fresh weight. DM dry matter; a, rapidly degradable fraction in rumen degradation; b, slowly degradable fraction in rumen degradation; c, the degradation rate of the slowly degradable fraction; ED, effective degradability of the incubated samples. Means in a row with similar superscripts do not differ (*p* > 0.05); SEM, standard error of means.

**Table 3:** Alpha diversity of fresh waxy corn stalk silage after 45-day fermentation.

Items	Treatment				SEM	*p*
	CON	ML	LE	MLLE		
Shannon	2.39 ^A^	1.84 ^B^	1.98 ^AB^	1.30 ^C^	0.143	<0.01
Richness	267.20 ^A^	200.00 ^B^	210.00 ^B^	154.40 ^C^	13.820	<0.01
Chao1	292.85 ^A^	216.07 ^B^	228.05 ^B^	176.31 ^B^	17.118	<0.01
Ace	294.43 ^A^	218.99 ^B^	228.49 ^B^	180.09 ^B^	17.503	<0.01

CON, control; ML, *Lactobacillus plantarum* at 1.0 × 10^5^ cfu/g fresh weight and *Bacillus subtilis* at 1.0 × 10^5^ cfu/g fresh weight; LE, lignocellulolytic enzyme system at 500 g/t fresh weight; MLLE, *Lactobacillus plantarum* at 1.0 × 10^5^ cfu/g fresh weight, *Bacillus subtilis* at 1.0 × 10^5^ cfu/g fresh weight, and lignocellulolytic enzyme system at 500 g/t fresh weight. Means with different uppercase superscripts within a column are significantly different (*p* < 0.05); SEM, standard error of means.

## Data Availability

The data presented in this study are available upon request from the corresponding author.
